# Family Businesses and Adaptation: A Dynamic Capabilities Approach

**DOI:** 10.1007/s10834-018-9586-3

**Published:** 2018-08-17

**Authors:** Abel Duarte Alonso, Seng Kok, Michelle O’Shea

**Affiliations:** 1 Liverpool Business School, Liverpool John Moores University, Redmonds Building Brownlow Hill, Liverpool, L3 5UG UK; 2 School of Business and Law, Edith Cowan University, 270 Joondalup Dr., Joondalup, 6027 WA Australia; 3 School of Business, Western Sydney University, Locked Bag 1797, Penrith South DC, NSW 2571 Australia

**Keywords:** Family businesses, Dynamic capabilities approach, Firm adaptation, Adaptive strategies, Western Australia

## Abstract

The main objective of this research was to propose a framework centred on the dynamic capabilities approach, and to be applied in the context of family businesses’ adaption to their changing business environment. Data were gathered through interviews with ten FBs operating in Western Australia. Based on the findings, the clusters of activities, sensing, seizing, and transforming emerged as key factors for firms’ adaptation, and were reinforced by firms’ open culture, signature processes, idiosyncratic knowledge, and valuable, rare, inimitable and non-substitutable attributes. Thus, the usefulness of the proposed framework was confirmed. Implications and future research opportunities are presented.

## Introduction

Past and current research documents the significance of family businesses (FBs) for different economies (e.g., Astrachan and Shanker 2003; Chirico and Nordqvist 2010; Howorth et al. 2010). Family businesses have been defined in various forms, for instance, as those firms where 51% or more is controlled by a family (Dumas 1992; Rosenblatt et al. 1985), and where family members influence key operating plans and decisions for the succession of their leadership (Handler 1989). A broader definition proposed by Poza and Daugherty (2014) and adopted in this study suggests that family businesses constitute a whole range of enterprises, where either a next-generation CEO or an entrepreneur, together with one or more members of the family can have a strategic influence on the firm. Furthermore, these individuals exert such influence through various means, including ownership control, board or managerial participation, or through values and culture as imparted by family shareholders to the firm (Poza and Daugherty 2014). Lastly, FBs comprise family members who bring resources together to attain specific goals for the business (Lee and Marshall 2013).

Poza and Daugherty (2014) explained that at least 80% of business in Asia, Europe, Latin America, and United states are owned and/or controlled by families. Furthermore, in the world’s most advanced economies, FBs account for the majority of employment and contribute to over 50% of countries’ gross domestic product (Poza and Daugherty 2014).

Past research (Chirico and Nordqvist 2010) has found that FBs face significant difficulties, including the transition between family generations, which prevents many FBs from surviving past their first generation (Dalpiaz et al. 2014). Similarly, it has been contended (Miller and Breton-Miller 2006) that the inclusion of numerous or later family generations can result in succession difficulties, some of which can take the form of political conflicts, or drain on family resources. Studies have identified additional hurdles, notably, in the form of difficulties in being able to raise optimal levels of financial capital (Memili et al. 2015), limitations in attracting and retaining highly qualified managers, and lack of an effective structure and limited networks (Sirmon and Hitt 2003). Finally, Ramírez Solís et al. (2017) have concluded that many FBs also face the dilemma of remaining competitive and maintaining their growth in a business environment which experiences rapid changes.

Various authors (Benavides-Velasco et al. 2013; Prencipe et al. 2014; Priem and Alfano 2016; Sharma and Chua 2013; Xi et al. 2015; Zahra 2016) have agreed that the body of knowledge of family business (FB) research has grown and is expected to increase. At the same time, others have identified limitations—and therefore future research opportunities—in the field of FB. First, while contemporary research has begun to elucidate the paradox of innovation among FBs, that is, research revealing positive and negative associations between family firms and innovation, overall, findings have been inconclusive (Duran et al. 2016). Second, limited research has focused on the interface of FBs and organisational behaviour (Sharma et al. 2014). Third, the field of FB has yet to become integrated into organisational science disciplines (Gedajlovic et al. 2012). Fourth, it has been suggested that more effort is required to understand the complexity of FBs, and how they may differ from—or be similar to—other business entities (Benavides-Velasco et al. 2013).

Fifth, while the FB literature has presented numerous competing theoretical frameworks, these characteristically have lacked empirical support (Zahra 2016). Sixth, limited research has critically examined the impact that the external environment has on FBs (Wang 2016). Finally, as stated by Fletcher et al. (2016) “the full potential of qualitative inquiry (in FB research) is not being fully realized” (p. 8).

This study was specifically concerned with these last three knowledge gaps, particularly in the context of FBs operating in rapidly changing business environments (Ramírez Solís et al. 2017). Moreover, through empirically investigating FBs, the study addressed the gaps recognised by Fletcher et al. (2016), Wang (2016) and Zahra (2016). Qualitative data were gathered, primarily through face-to face interviews with 10 FBs operating in Western Australia, five of which were involved in international business activities. Overall, the study sought to address the following overarching research question (RQ):

RQ1: How do FBs adapt to a rapidly changing business environment? For instance, what specific resource(s) are FBs leveraging to remain competitive in such environment?

According to Gedajlovic et al. (2012), examining FBs can provide valuable insights to questions and issues “with which mainstream management scholars are currently grappling” (p. 1011). At the same time, and in line with the focus of this study, a theoretical framework underpinned by the dynamic capabilities approach (DCA) (e.g., Eisenhardt and Martin 2000; Teece et al. 1997) was proposed. Indeed, this approach was especially appropriate to study the business practices of firms facing a dynamic business environment (Teece et al. 1997). Incorporating the DCA allowed the study to address yet another research gap, notably, the limited empirical research implemented to understand DCs in the context of family firms (Wang 2016). Therefore, the following additional RQ was also investigated:

RQ2: How is the DCA manifested in the context of the participating FBs?

## Background and Conceptual Model

### Family Businesses, Adaptive Capabilities and Resilience

The focus of this research on adaptability provided strong justification and emphasis on the importance of instrumental ways through which FBs build adaptive capabilities and resilience. Chirico and Salvato (2008) postulated that the high-speed characteristics of competitive business environments has induced many firms to recognise the essential role of “enablers of dynamic organizational adaptation” (p. 169) for firms’ sustainable competitive advantage. This notion is particularly significant among family firms who face specific threats to survive or to achieve transgenerational success (Chirico and Salvato 2008).

Some studies have suggested the nexus between family firm members and the development of adaptive instruments for firms to adapt and become more resilient. For example, McDonald and Marshall (2018) explained that FBs comprise a complex network of resource exchanges and interpersonal relationships. In line with Chirico and Salvato (2008), McDonald and Marshall (2018) also put forward that these relationships and exchanges are central to long-term firm sustainability or even to their short-term success. FBs also rely heavily on resource allocation, which represent “a continuum of possible allocation decisions” (p. 165) that range from the use of firms’ profit (e.g., investing back on the business, to allocating it for family savings or consumption. paying taxes).

Other mechanisms have been suggested to have important implications for FBs’ competitiveness, and therefore play a key strategic role in allowing them to become more adaptive and resilient. In fact, Lee and Marshall (2013) found that FB owners’ goal orientation, measured through positive reputation among their customer base or through firm growth, were significant and had a positively effect on FB performance. Along these lines, FB research by Hatum and Pettigrew (2004) revealed the role of the firm’s founder, especially in instilling a strong identity, which rested on core values shared throughout family generations, were also valuable in significantly motivating and facilitating change. Symbolic associations in the form of emotional attachment, together with moral obligations and values within the family business, have also been identified as factors to carry on and continue with the FB (Glover 2010).

By extending this body of knowledge, this study was conducted with family firms operating in Western Australia, and has made several important contribution to the extant FB literature. Fundamentally, it helped narrow several knowledge gaps presented in previous studies (Fletcher et al. 2016; Wang 2016; Zahra 2016), examining family firms through the lens of a theoretical framework and based on a qualitative research approach. Moreover, by proposing a theoretical framework based on the DCA and in the context of FBs, the study has made a significant theoretical contribution. The DCA is discussed in the next section.

### Dynamic Capabilities (DCs) and the DCA

DCs have been defined as firms’ ability to build, reconfigure, and integrate external and internal competences and be able to respond to the rapidly changing business environment (Teece et al. 1997). The term “dynamic” underscores firms’ “capacity to renew competences” (Teece et al. 1997, p. 515) and achieve congruence within their changing business setting. Capabilities highlights the influence of strategic management in reconfiguring, adapting, and integrating external and internal resources, functional competences and organisational skills and match the demands of their business environment (Teece et al. 1997). Furthermore, capabilities have been perceived as substantially “home-grown” key organisational elements, enhancing firms’ capacity to perform, thus, conferring inimitability and, consequently, enhancing competitive advantage (Helfat and Winter 2011).

Eisenhardt and Martin (2000) stated that DCs represent a set of identifiable and specific processes that entail alliancing, strategic decision making, and product development. These processes are experiential, unstable, and simple; they are based upon new knowledge, embedded in firms, and exhibit commonalities that can be referred to as firms’ best practice, helping them to be adaptive (Eisenhardt and Martin 2000). In fact, DCs encompass adaptation and change, as they reconfigure, integrate, or build other capabilities and resources (Helfat and Peteraf 2003).

While DCs are necessary for firms to achieve, as well as continually leverage and enhance competitive advantage, they are not sufficient (Eisenhardt and Martin 2000). To qualify as sources with competitive or sustained competitive advantage, firms must possess various key attributes, often referred to as VRIN, or valuable, rare, imperfectly imitable/inimitable, and un-substitutable (Eisenhardt and Martin 2000). Thus, the DCA is an extension of the resource-based view (RBV) of the firm (e.g., Ambrosini and Bowman 2009; Ambrosini et al. 2009), a theory which emphasises the strategic significance of those key attributes (Barney 1991, 2001a, b).

Aligned with the definition of DCs, the DCA analyses the firm’s methods and sources of wealth capture and creation among firms operating in rapidly changing and increasingly ambiguous and demanding business settings (Teece et al. 1997). Furthermore, the DCA proposes an articulated framework which can integrate empirical and conceptual knowledge, thereby facilitating prescription (Teece and Pisano 1994).

Since its initial development in the 1990s, the theoretical foundation of the DCA has been strengthened by more recent contributions. For example, Teece (2007) explains that DCs support such enterprise-level capacities as sensing, seizing, and transforming; these capacities are complex to deploy or develop. These clusters of adjustments and activities (Teece 2012), or orchestration processes (Teece 2014a) have been conceptualised as follows:

1. *Sensing*—also associated with *shaping*—new opportunities and threats (Teece 2007) comprises identification and assessment of opportunities (Teece 2012). Thus, sensing entails scanning, interpreting, creating, and learning (Teece 2007), and is complemented by investment in research (Teece 2007).
2. *Seizing* When market or technological opportunities are sensed, they must be addressed through new processes, services, or products (Teece 2007). This process of mobilising resources (Teece 2012), often demands investments in such activities as commercialisation and development (Teece 2007).
3. *Reconfiguring or transforming* In order to sustain growth, firms must have the ability to transform or recombine their organisational structure and assets (Teece 2007). Moreover, as technologies and markets change, these continuous renewal activities must be executed expertly (Teece 2012).

A subsequent contribution reaffirms the importance of these three clusters of activities as a first step for firms to achieve competitive advantage. Indeed, in proposing a logical structure of the DCs paradigm, Teece (2014a) theorised that organisational heritage and managerial decisions, which are grounded on the above clusters of activities, are linked to DCs and firm resources (VRIN attributes). These theoretical constructs are also in accord with Teece and Pisano (1994), who posited that competitive advantage derives from DCs engrained “in high performance routines operating inside the firm, embedded in the firm’s processes, and conditioned by its history” (p. 553).

However, firms also need good strategy (Teece 2014a). Consequently, a framework proposed by Teece (2014a) illustrates two-way associations with ordinary capabilities, generic resources, and, importantly, with the need to build DCs and VRIN-related resources. Teece’s (2014a) conceptualisation was associated with Teece and Pisano’s (1994) earlier work, which underscores “the non-tradability of ‘soft’ assets” (p. 553) or capabilities. These non-tradeable assets include firms’ culture, values, and organisational experience. Generally, these assets cannot be acquired; they need to be created (Teece and Pisano 1994).

### The DCA in the Context of FBs

Research by Benavides-Velasco et al. (2013) highlighted the suitability of both RBV and DCA “as… theoretical perspectives to advance… family business research” (p. 55). DCs have been referred to in various FB-related studies (e.g., Cucculelli et al. 2014; Jones et al. 2013). Concerning adaptation, Jones et al. (2013) examined the associations between entrepreneurial cognition, multigenerational ownership, and DCs using the case of Liverpool’s only surviving family-owned shipping firm.

Some of the DCs that emerged in Jones et al.’s (2013) research were interlinked with diversification, and included leveraging existing company (marine) resources, developing new resources, such as retail, distribution and financial activities. A selection of verbatim comments gathered by Jones et al. (2013) revealed specific ways that contributed to the company’s adaption to the new challenges of its changing business environment. In particular, adaptation in the form of adjusting plans to the company’s circumstances, exploiting opportunities, engaging in a specific strategic vision, as well as increasing autonomy and overall empowerment of management were manifestations of DCs (Jones et al. 2013).

With regard to change, Chirico and Nordqvist (2010) investigated the relationships between DCs and trans-generational value creation among FBs. These authors explained that any capability comprises static and dynamic components, which, in the face of the changing business environment, and depending on firms’ organisational culture, may result in change or inertia. They also posited that family inertia, which depends on the FB’s culture can prevent or stimulate the development of DCs.

Chirico and Nordqvist (2010) proposed a framework which highlighted the importance of (idiosyncratic) knowledge and its association with DCs, which in turn can affect FBs’ performance, and subsequently have an impact on firms’ transgenerational value. Importantly, the authors emphasised the importance of tacit knowledge, or unarticulated knowledge that includes implicit rules of thumb, physical experiences, or intuition (Nonaka and Von Krogh 2009). Moreover, by working in the FB early on in their lives, family members can develop an in-depth level of firm-related tacit knowledge (Chirico and Nordqvist 2010).

Chirico and Nordqvist (2010) also hypothesised that transgenerational value can be a vehicle allowing FBs to make further investments and to acquire more nuanced knowledge, notably, through training, or by employing external staff. Therefore, knowledge, DCs, and entrepreneurial performance constituted the initial part of Chirico and Nordqvist’s (2010) theoretical framework.

The second part was based on these authors’ own findings from investigating four family firms. On one hand, they noticed that FBs’ organisational culture had strong impacts on how the ownership/management perceived change. Furthermore, when FBs displayed a closed, paternalistic culture, their attitude leaned towards not making changes. Instead, FBs tended to make autonomous changes and choices, and exhibited limited freedom with regard to expressing ideas (Chirico and Nordqvist 2010). On the other hand, those FBs exhibiting an open culture demonstrated entrepreneurial orientation through their attitude to making changes, embracing proactiveness and innovativeness and risk-taking (Chirico and Nordqvist 2010), thereby illustrating links with DCs. Consequently, the authors observed the significance of the dynamic dimension of capabilities in helping to avoid rigidity and become trapped.

Jones et al. (2013) recognised that the empirical work by Chirico and Nordqvist (2010) appeared to be the only one applying the DCs framework to examine FBs. Nevertheless, these two studies clearly documented the considerable merit of examining FB adaptation and change through the lens of DCs. With these notions in mind, this study has utilised the DCA in the development of a new theoretical framework.

### Proposed Theoretical Framework

**Fig. 1:**
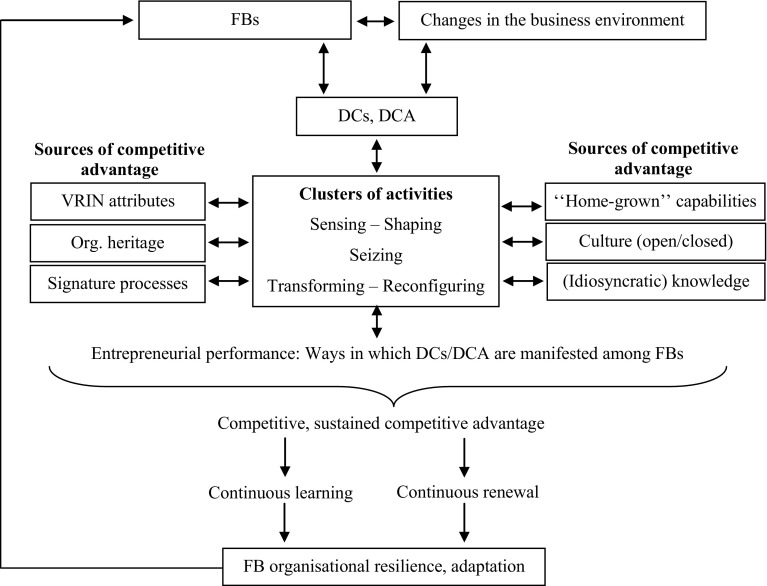
The DAC in the context of FBs. Reproduced with permission from Chirico and Nordqvist (2010), Gratton and Ghoshal (2005), Helfat and Winter (2011), Teece (2007, 2012, 2014a, b), Teece et al. (1997)

Based on the previously discussed DCA literature, a theoretical framework associating DCs and FBs (Fig. 1) is proposed in this study. The framework first hypothesises strong links between FBs, the changing business environment, DCs and the DCA. In turn, these elements are interlinked with the clusters of activities, or sensing, seizing, and transforming (Teece 2007, 2012, 2014a) and sources of competitive advantage. Teece (2014a) argued that, typically, DCs cannot be acquired, are difficult to imitate, and are built within the organisation. This level of uniqueness and inimitability is based on firms’ VRIN resources, signature processes, and are a result of past managerial decisions and heritage, and include (managerial) actions, context-specific learning, or investments (Teece 2014a).

Gratton and Ghoshal (2005, p. 49) referred to “signature” as “a company’s character” and “idiosyncratic nature,” and further explained that signature processes occur from interests and passions within the firm. As Teece (2014a, b) suggested, these processes have deep roots, and, because they entail specific values or history, other firms cannot replicate them easily. In this context, and in accord with Helfat and Winter (2011), the strategic value of “home-grown” capabilities is also perceived as fundamental for firms to address changes in their business environment. FB research (Distelberg and Blow 2010) identified that unifying values across members of the FB was a strong contributor of satisfaction.

Similarly, the framework recognises strong links between Chirico and Nordqvist’s (2010) theoretical contribution and the clusters of activities. Moreover, Fig. 1 considers the perceived impact that knowledge has on FBs, as well as the authors’ findings revealing the influence of organisational culture within FBs, particularly attitudes regarding the implementation of change processes.

Further in agreement with Chirico and Nordqvist’s (2010) research, the importance of entrepreneurial performance is theorised as a result of all strategic elements, or sources of competitive advantage (e.g., VRIN attributes, the organisation’s heritage, culture, and knowledge). Entrepreneurial performance is illustrated, in part, by the avenues in which DCs and the DCA are manifested within the participating firms. These avenues have implications for firms’ competitive and sustained competitive advantage. Consequently, the framework emphasises the need for firms to build upon their signature processes, “home-grown” capabilities, and other strategic processes and practices, as they continue their journey of continuous learning and renewal to adapt to changes in their business environment and build organisational resilience.

## Method

The present empirical study adopted the DCA and proposed a framework based on this approach to examine ways in which FBs adapt to the changing business environment. Therefore, the study’s unit of analysis, or the notion of a conjoint set of components that comprise the entity at the centre of the research (Gronn 2002, p. 444) was concerned with identifying the resources and capabilities, including DCs, FBs possess and operationalise in order to adapt. The thrust of the research, which emphasised the identification of themes related to the research questions, justified the choice of an inductive analysis. This approach primarily employs meticulous interpretations of raw data allowing researchers to derive themes, concepts, or a model based on that raw data (Thomas 2006). The approach also constitutes a methodical set of actions needed to analyse qualitative data (Thomas 2006), which can produce valid and reliable findings. With an inductive approach, the researcher starts with specific data that are employed to develop—or induce—a broad “explanation (theory) to account for the data” (Engel and Schutt 2005, p. 45).

A case study method, referred to as a research strategy focusing on the understanding of dynamics that are present within a single setting (Eisenhardt 1989) was utilised in this study. Case studies are preferred when (1) a contemporary phenomenon in its real context is the main focus, (2) the researcher has limited control over events, and (3) when how or why questions are asked (Yin 2009). The associations between Yin’s (2009) suggested criteria and this study justified the decision to choose a case study methodology. Essentially, the study examined a contemporary phenomenon, notably, adaptation in the context of the participating firms’ business environment. Furthermore, the researchers in the study did not have any control over the events affecting firms. Finally, the study fundamentally asked a “how” question.

Typically, case studies can incorporate various data collection methods, such as interviews, observations and archives (Eisenhardt 1989). In this study, these three forms of data collection were employed. Data were gathered through face-to-face interviews, complemented by on-site observations, and finally by document analysis to include website information and printed organisational materials. As Muske and Winter (2001) posited, the case study approach depends on in-depth interviews; these allow the researcher to understand the experiences of individuals, and the meaning of such experiences to them. In addition, as Danes et al.’s (2016) research carried out among families residing in the United States acknowledged, qualitative interviews help investigate contextual processes, and reveal dynamic processes. Nonetheless, it is also important to note the value, content and context provided by observations and document analysis.

Given the need to select individuals who had an in-depth knowledge of the participating FBs, including family members with experience growing up or working in the firm, a purposive sampling approach was selected. This methodology entails the selection of the most valuable sample to answer research questions (Marshall 1996). Moreover, purposive sampling is most effective when researchers need to investigate particular cultural domains with individuals within these who are knowledgeable experts (Tongco 2007). Palinkas et al. (2015) identified various types of sampling, including extreme/deviant, critical cause, maximum variation and intensity sampling. This last form entails information-rich cases where the phenomenon of interest is manifested intensely (Patton 2015). Thus, the characteristics of the sampling for this research, which are strongly based on information-rich cases, fall under the intensity sampling category.

Between June and August of 2015, the owners and managers of 17 family businesses operating in Western Australia, where part of the research team was based, were contacted. The geographic context for conducting the research, which was based in the state of Western Australia, also allowed opportunities to learn more about FBs in this state, where a very limited number of studies on FB adaptation have been conducted. The 17 FBs were identified through desk research, particularly by using online resources, including from business associations, news reports, and individual company website information.

The identified firms were subsequently contacted, informed about the study’s objectives, and formally invited to partake in the research. A total of 10 businesses positively responded to the research team’s invitation. From July 2015 and February of 2016, and based on their availability, the owners/co-owners of nine and the manager of one of these businesses were interviewed. Although this last individual was not a family relative of the firm’s ownership, she had accumulated 21 years of work experience as a financial manager and worked her way up within the company, which allowed her to learn from different perspectives throughout her journey. In addition, she had regular direct contact with members of the family firm over the many years of her tenure, and was designated by the management to speak on behalf of and represent the company during the interview. Overall, her strong knowledge and expertise were very valuable to the research, thus, justifying her inclusion. Similarly, her comments and opinions would strongly reflect the views of the family.

Semi-structured, face-to-face interviews were conducted with participants, which allowed for gathering printed company information and for making observations of the premises. On average, the interviews lasted 75 min, and were complemented by email communication in the months following the interviews, for instance, to gather updates. The interviews started with several questions designed to collect demographic data, for instance, the age of the FBs alongside the background and experience of participants in the FB. Subsequently, in considering several FB academic contributions (Allison et al. 2014; Chirico and Nordqvist 2010; Dalpiaz et al. 2014; Duran et al. 2016; Howorth et al. 2010), including those focusing on FB adaptation (Chirico and Salvato 2008; Hatum and Pettigrew 2004; Poza and Daugherty 2014), the following questions were asked:

- How does your firm adapt to today’s business environment?
- In what ways does it adapt?
- What specific resource(s) does the FB exploit to address the changing environment in which they operate?

According to Marshall et al. (2013), data saturation, reached when data are gathered until no new information is added (Bowen 2008), “is an elusive concept and standard in qualitative research since few concrete guidelines exist” (p. 11). Not surprisingly, the adoption of saturation as a universal quality indicator is inappropriate (O’Reilly and Parker 2013). Instead, the importance of sample adequacy should be emphasised; moreover, sample adequacy is not to be determined by the number of respondents but instead by the appropriateness of the collected data. Aligned with O’Reilly and Parker’s (2013) notion, in this study such appropriateness was noticed by the 10th interview, when no new emerging themes, patterns or threads were noticed. Similarly, recurrent issues reflected consistency within the dataset, suggesting the identification of key findings and verifying the appropriateness of the data collected through the three different sources.

In order to maximise consistency and transparency, the interview data were transcribed and cross-checked by members of the research team. The data were analysed employing qualitative content analysis (QCA), which Schreier (2012, p. 1) defined as a method that is employed to describe the meaning of qualitative data systematically, and that consists of classifying content “as instances of the categories of a coding frame” (p. 1). Moreover, QCA often involves more subjective, broader code categories, whereby the data are used as the coding source (Morgan 1993).

Consequently, QCA has precise characteristics. First, as a method QCA emphasises context and subject, as well as similarities and differences between categories or codes (Graneheim and Lundman 2004). Second, QCA deals with latent as well as manifest content in text data. Latent content has been perceived as themes, or what the text refers to, while manifest content is what is written in the text, and it is typically presented in categories (Graneheim and Lundman 2004). The procedures followed in this study conformed to the definitions and characteristics of QCA. In fact, aligned with Morgan (1993) and Schreier (2012) the data collected were analysed based on the authors’ interpretation, and in ways to describe meanings; these were classified and broken down into categories (Table 2).

Furthermore, and in accord with Graneheim and Lundman (2004), the data collected also featured the characteristics presented by Graneheim and Lundman (2004), notably on the emphasis of subject and context, as well as the manifested and latent content that were illustrated both in Table 2 and in selected verbatim comments from participants.

Complementing this process, NVivo, version 11, a computer-assisted qualitative data analysis software (Cope 2014), was employed. This software assisted in identifying clustering and grouping of themes across the three different sources of data.

### Demographic Characteristics of Participants

Table 1 illustrates some demographic characteristics of participants and their FBs. Nine of them directly owned or co-owned the FB at the time of the interview. While there was variety regarding the industry in which firms operated, a predominance of firms involved in different forms of food production, particularly fresh produce, was noticed. Indeed, six firms produced foods, including vegetables, cattle, and fruits (Tim, Sam, Dan, Marie, Rose, and John). In addition, four firms (Jennie, Tim, Sam, John) were exporting at the time of the study, three (Dan, Marie, Rose) had previous exporting experience, and one (Nick) was involved in imports. The age of the firms ranged between more than a century (Ely’s company) and six years (Nick), with seven being part of at least the second family generation.

**Table 1:** Demographic characteristics of HRC members

P^a^	Participant’s role	Industry	Life of the business^b^	Size (full-time employees)
Jennie	Owner	Packing/exporting avocados (1st generation)	12	30
Tim	Owner	Fresh produce (3rd generation)	78	100
Ely	Co-owner	Food supplier (4th generation)	122	200
Sam	Owner	Fresh produce (2nd generation)	70	100
Dan	Co-owner	Fruit grower (2nd generation)	41	3
Marie	Co-owner	Fresh produce (2nd generation)	28	35
Rob	Owner	Designs (e.g., glass windows; 2nd generation)	60	40
Rose	Manager	Food manufacturing (e.g., hams)^c^	66	400
John	Co-owner	Cattle (4th generation)	14	0
Nick	Co-owner	Coffee, tea imports (1st generation)	6	0

^a^Pseudonyms were used to label participants (e.g., participant 1: Jennie)

^b^Age of the business (given in years) at the time of the interview

^c^Currently, family-controlled but no direct family involvement in this firm

Similarly, there was a significant gap regarding firms’ sizes, with three employing three or no staff, and one (Rose) as many as 400. Based on the Australian Bureau of Statistics’ (ABS 2001) definitions of firms, three are considered micro in size (less than 5 employees), five medium (between 20 and 199 employees) and two large (200 and more employees). Finally, four business were in their second family generation, two in their first and fourth, respectively, and one in the third generation. Regarding Rose’s case, though family-owned for numerous decades, and family-controlled at the time of the study, there was no direct involvement of family members in managerial positions.

## Results and Discussion

### FBs’ Adaptation to the Changing Business Environment and Ways to Adapt

The content analysis undertaken (Table 2) helped summarise the changes participants perceived in their business and industry, how they were adapting, and ways in which DCs and the DCA were manifested. Overall, numerous associations between the findings and the DCA were noticed. Furthermore, and as discussed in the following section, in multiple instances the findings were aligned with the proposed framework (Fig. 1).

**Table 2:** Qualitative content analysis: emerging themes and categories

P^a^	Changes (business environment)	Ways to adapt (RQ1)	Manifestation of the DCA among FBs (RQ2)	
			Clusters of activities (DCs)	DCs-related key elements
Jennie	Increase in product demand	Innovating, diversifying, entering new consumer markets/sectors, networking	Sensing, seizing, transforming	“Home-grown” capabilities, VRIN attributes, culture (open), signature processes, idiosyncratic knowledge
Tim	Increase in product demand	Innovating, entering new consumer markets	Sensing, seizing, transforming	Organisational heritage, “home-grown” capabilities, VRIN attributes, culture (open), signature processes, idiosyncratic knowledge
Ely	Transformation of the industry	Innovating, strengthening consumer relationships	Transforming	Organisational heritage, “home-grown” capabilities, VRIN attributes, culture (open), signature processes, idiosyncratic knowledge
Sam	Increase in product demand	Increasing exports, innovating, adding value	Sensing, seizing, transforming	Organisational heritage, “home-grown” capabilities, VRIN attributes, culture (open), signature processes, idiosyncratic knowledge
Dan	Industry decline	Diversifying	Transforming	Culture (open), signature processes, idiosyncratic knowledge
Marie	Increase in product demand, legislation	Innovating, adding value, networking	Sensing, seizing, transforming	Organisational heritage, “home-grown” capabilities, VRIN attributes, culture (open), signature processes, idiosyncratic knowledge
Rob	Legislation, consumer demands	Ensuring compliance, innovating, increasing knowledge	Sensing, seizing, transforming	Organisational heritage, “home-grown” capabilities, VRIN attributes, culture (open), signature processes, Idiosyncratic knowledge
Rose	Industry, consumer demands	New product development, adding value	Sensing, seizing, transforming	Organisational heritage, “home-grown” capabilities, VRIN attributes, culture (open), Idiosyncratic knowledge
John	Industry, increase in product demand	Innovating/adding value, entering new consumer markets (exports), networking	Sensing, seizing, transforming	Organisational heritage, Idiosyncratic knowledge, signature processes, “home-grown” capabilities, VRIN attributes, culture (open)
Nick	Consumer demands, interests	Increasing networks, knowledge	Sensing, seizing	Idiosyncratic knowledge, signature processes

^a^Pseudonyms were used to label participants (e.g., participant 1: Jennie)

In five cases, participants’ comments suggested that the need for adaptive measures had been triggered by changes in the market, particularly through greater consumer demand (Table 2). For example, Jennie’s case, documented through on-site observations and the interview, demonstrated how the FB transformed from being an avocado producer to becoming a packing and exporting firm as a result of identifying—and anticipating—a future growth and at present untapped commercial opportunity. As Jennie explained, an increase in the number of avocado producers in recent years resulted in an excess of supply, exposing the region’s weaknesses of lacking appropriate logistics foundation and infrastructure, including industry expertise, and, more urgently, a packing facility to absorb and streamline rapid growth. Jennie’s company used its accumulated knowledge and previous experience to become a consulting firm to the local avocado producers. In this process, the firm turned to innovative initiatives, and significantly invested in equipment and technologies. For example, through the development of an application for growers to monitor their production needs, the FB helped them achieve efficiencies while facilitating production processes. The participant also identified new trends and consumer wants that led to new product development, including a baby food line and food for aged care facilities, thereby maximising food production:

We’re also doing a baby food line… you utilise all of those vegetables and fruits. They all come out as purees that you can turn into baby food… in the food service options, I sort of became aware of a bit of a raw deal that aged care facilities get in Australia. So I made it a bit of a mission that I wanted to do something about that…

Establishing networks with other producer associations to begin exports, or to help the FB develop its baby food project was also an essential element differentiating this firm: “Although I’m in the avocado industry, I actually know people in lots of industries, across the board…” This finding found support in the contemporary literature, with Zheng et al. (2011), positing that networks and alliances have become a key part of firms’ business environment in recent years. Through their research, these authors also found that network embeddedness acted as a key antecedent of DCs. In this context, Zheng et al.’s (2011) research revealed the importance of relational embeddedness, which highlights the features of direct ties, notably, in promoting extensive and deep exchange of knowledge.

Jennie’s case also illustrated that, if properly operationalised, different demands, needs and wants in firms’ business environment could be turned into unique commercial opportunities. Notably, in line with Chirico and Nordqvist (2010), Jennie’s firm exhibited an open culture to changes, valuing the contributions of all of the firm’s members: “*What’s important is that team element, and realising that everyone has different strengths and weaknesses, but as a group we’re quite strong because we back each other*.” The firm’s open culture, together with idiosyncratic knowledge of the operational side of the industry, as well as knowledge of significant stakeholders, signature processes, home-grown capabilities and VRIN attributes, clearly represented sources of competitive advantage.

The cases of Tim, Sam, Marie and John also demonstrated similar characteristics. For example, having experienced currency fluctuations that had affected the FB’s earlier exporting endeavours, Tim underlined the importance of sensing and seizing (Teece 2007). After focusing on the wholesale market, Tim made a conscious decision to research the international market, and was able to identify lucrative commercial opportunities. Tim’s comments also revealed facets of sensing, seizing and transforming when he explained the FB’s current and future strategies to adapt to the changing fresh produce environment:

We grow about twice as much product as what we need per week, because every week we’re selling products somewhere. Business is quite different, [so] you’ve got to make sure that you have enough product to supply everybody you know…

Sensing and transforming also became evident when the participant commented on a proposed trip to Germany to observe new fresh produce growing techniques, and when he anticipated future trends in consumer demands: “*The next form for value adding corn would be partially cooking*.”

Partly aligned with Tim’s case, Sam’s firm was experiencing the rapidly changing fresh produce consumer environment. After buying over the firm, which had gone through receivership, Sam had worked to increase the FB’s involvement in international exports. At the same time, he focused on decreasing wholesales to domestic supermarkets, thereby adding vital value to its food production. Sam explained that the firm now sold as much domestically as internationally, which represented a notable development, given that prior to this change 75% of Sam’s production was sold domestically.

Through this experience, Sam learned the importance of becoming less dependent on large supermarkets: “*We wore the agricultural risk… the economic risk, all the commercial risk as well… and I just didn’t see the point*.” While increasing exports also had risks, including those associated with currency fluctuations, the firm had accumulated industry knowledge and expertise to implement key strategies:

We’re probably a little bit unique, we sell everything at a fixed price to all our customers, domestic and exports, so we wear the agricultural risk but our customers wear the currency risk. And that’s across all markets…everyone buys in Aussie dollars.

As was the case of Jennie’s enterprise, the evolution of Sam’s firm in supplying fresh produce to demanding but lucrative consumer markets also required technology and equipment related innovations, again, with the need for making significant investments, especially financially and time-wise: “*We did spend a lot of time getting the cold chain right and that’s probably the key part of our business. It does come at a cost though, between electricity and equipment costs…but it’s definitely been worth it*.” Innovating and differentiating from competitors was also demonstrated in the firm’s commitment to use more environmentally-friendly energy sources: “*70–80% of all our energy needs come from wind [-generated energy]*.” Sam’s experience illustrated the importance of process innovation, or the introduction of new—or considerably improved—production, administrative, or supply chain processes (Piening and Salge 2015). Process innovation is one key source “of competitiveness in dynamic industries” (Piening and Salge 2015, p. 80).

Despite the FB’s investments and efforts, Sam acknowledged the highly competitive environment of some export markets, and while being *“clean and green, the reality is if something is materially cheaper from another country, they [consumers] will by it*.” The ability to sense the environment and anticipate potential challenges ahead has persuaded the firm to make a progressive shift in export focus. For example, and as with Tim’s case, Sam recognised the potential of other markets: “*Definitely the Middle East. That’s the one that’s taken off the most in the last couple of years. Asia’s becoming harder*.” Accordingly, this finding was in part related to Nonaka and Von Krogh’s (2009) suggestion that intuition and tacit knowledge greatly influence FB’s strategic business decisions as is the case of Sam’s investments and involvement in various export markets.

Other firms (Marie, John) were also experiencing an increase in demand for their products, and had perceived the potential for value adding through their product offerings, which again illustrates an alignment with seizing and transforming (Teece 2007). Marie acknowledged the long family history and tradition in the fresh produce industry, which rested on high quality and reliability, and helped build and maintain a positive reputation and respect among its clients. With the increase of local demand for fresh produce during the years of Western Australia’s mining boom and the growth of Perth’s population, Marie’s firm made a strategic decision to produce throughout the year as opposed to seasonally. However, in more recent years, the Western Australian mining industry has experienced a downturn, and other firms entered Marie’s industry, negatively affecting the company’s anticipated future growth. To adapt to this change, and in accord with Zheng et al. (2011), the importance of existing relationships with market agents increased, with Marie’s firm starting to add value to its products. Indeed, through recommendations from their sales agents, the FB changed its packaging and started barcoding its products, which resulted in an increase in sales. Importantly, this case also underlined the nexus between industry networks and process innovation (Piening and Salge 2015).

The significance of adding value to food production and strong relationships with end consumers also became evident in John’s case. The participant recognised that his firm set itself apart from many other farming operations, in that from the outset, it employed direct marketing for its products and developed unique packaging for its meats. The FB’s long history in the industry, acquiring market knowledge, skills and critical insights helped the firm to respond to consumer trends, thereby sensing and seizing opportunities. For instance, the firm employed the strategy of only producing organic beef, a product with significant appeal for niche and lucrative markets.

Transformation was also revealed in the firm’s evolution, from selling domestically, to entering exports markets. In fact, the participant acknowledged making significant strides in this part of the business, starting in Singapore, and planning to continue exports to the Middle East. The focus on niche markets was also reflected in the firm’s involvement with local high-end clients, including a luxury hotel in Perth, and selling to people with whom John’s firm had established solid business relationships:

Generally the guys that we deal with, we’ve dealt with them for a long time. They know the product, they appreciate that they may pay a bit more for it, they recognise what it is, and they’re happy to work with us.

This finding was associated with network embeddedness (Zheng et al. 2011), as well as with the notion of supplier integration capabilities. In fact, research by Vanpoucke et al. (2014) identified the importance of this construct as a tool for competitive advantage. Moreover, they found that integration sensing, seizing and transforming conformed sub-capabilities that as a whole constitute a dynamic capability.

Another participant’s comments (Ely) identified the continuous transformation of her business during more than 120 years of existence, from being first a grocery store, then entering the fresh seafood sector to become a major supplier and wholesaler, and finally a logistics company. The knowledge and expertise accumulated over such a long time and through several family generations facilitated such transformation. For instance, Ely reflected on the significant recognition and numerous awards obtained, as well as on the company’s strengths, which constituted a strong foundation for its performance and sustained competitive advantage:

We can get products to customers wherever they may be…we process every day fresh fish and we’ll supply it with all our orders to restaurants, hotels and whoever it may be… We are known in the industry for providing high service. We have people taking telephone orders, so chefs, after they finish in the restaurants at night, before they close they can call us up to ten pm and place an order for next-day delivery.

The above comments illustrated strong relationships with all the elements interconnecting DCs and the DCA, including the firm’s organisational heritage, its (open) culture to change, home-grown capabilities, idiosyncratic knowledge and VRIN attributes. As with Marie, Ely’s case also highlighted the importance of the human component, or direct communication and highly personalised service with its clients, which also constituted a source of competitive advantage. “Seizing” was clearly reflected in the careful and systematic service provision, and was further complemented by reliable and unrivalled delivery options (Ely): “*Logistically… we have branches up north… our competitors don’t have that, so they can’t access the markets, [or] ships they don’t supply…*”

The firm’s provision of high quality and personalised logistic services is partly aligned with Rothaermel and Hess’s (2007) research, which suggested that when studying firm innovation and adaptation one needs to consider the firm’s intellectual human capital. This resource can take the form of highly talented and skilled employees, and has associations with tacit knowledge (Rothaermel and Hess 2007). Through the different transformations of its business focus for over a century, to become a leader in its industry, Ely’s FB had acquired crucial, difficult to imitate resources, such as its tacit and idiosyncratic knowledge, and, in accord with Chirico and Nordqvist (2010), developing an open culture to change.

The cases of Dan and Rose illustrated two FBs with previous exporting experience that were now adapting to a new business reality in different ways. While traditionally focusing on the state of Western Australia, Rose’s firm had made significant investments in recent years to adapt and have a stronger presence in other Australian markets:

…with most of the population of Australia on the east coast…for us and our product, which has a short shelf life, it’s a difficult supply chain to manage…that’s why we have a factory on the east coast, and we’re currently going through a process of establishing a bigger factory on the east coast, just so that we can get close to the market.

Additionally, in order to remain competitive against other larger manufacturers of meat products, Rose recognised that the firm strongly communicated and met with their international meat providers, often travelling internationally. The firm also accessed large quantities of data to gain more explicit knowledge of what consumers were buying. This learning process was particularly useful when developing new products, including new meat flavouring (e.g., honey, smoked), or products with salt and fat reduction.

Throughout his more than 40 years of experience in the stone fruit industry, Dan had noticed the continuous transformation of the stone fruit sector. Indeed, due to increasing labour costs and the increasingly high value of the Australian dollar, both fruit canning and exporting had experienced strong decline. Now near retirement age, and using his extensive knowledge of the region and state, the participant and his brother made a decision to diversify into tourism, acquiring a bus to provide tours to and outside the region. At the same time, during and after the harvesting season, the firm continued to add value to his stone fruit production by selling onsite, taking advantage of the orchard’s unique location next to a main road.

Being the second family generation, and operating in Western Australia’s north, hours away from Perth, Rob’s firm had accumulated valuable knowledge and expertise in the area of installation of glass materials (doors, windows, double glazing). However, as suggested in Table 2, changes in governmental policy and associated legislation, as well as consumer trends, challenged the firm and called for adaptive strategies: “*It’s a never-ending evolution of product enhancements that we’re seeing… over the last three to five years we’ve all had to start to comply with proper codes… So I’ve seen a real shift in compliance…*”

As a result, the firm’s management was compelled to learn and apply its knowledge as well as expertise to take on the challenge of compliance. Moreover, management were keen to invest in order to see and experience first-hand new products or designs. In this regard, Rob acknowledged that the firm played a leading role in its region:

…up to a about a decade ago nobody did [engage in research], but we started to travel to some of the industry events over in Germany and China and certainly saw what was happening around the globe and realised there was an opportunity to expand…

Again, the importance of sensing, seizing, and transforming was revealed in Rob’s case. By making crucial changes, notably, being up-to-date with compliance requirements, or investing in gathering key strategic knowledge, the firm differentiated itself from other, and, arguably, built its competitive advantage.

Finally, having owned an import business for less than a decade, Nick’s case demonstrates associations with signature processes (Gratton and Ghoshal 2005) idiosyncratic knowledge (Chirico and Nordqvist 2010), and network embeddedness (Zheng et al. 2011). In fact, the participant’s interest and passion for developing a coffee culture in Western Australia was usefully complemented by strong family links, notably, from his spouse’s side with international contacts: “*Our connections across the coffee network have been growing, sourcing from other suppliers, and the different ways that they do business*.” This strategic advantage became essential in competitively managing the logistics of importing coffee, saving crucial time and enabling the firm to navigate through complex regulations and paper work requirements in the country of origin.

## Conclusion

Despite encountering criticism (e.g., Arend and Bromiley 2009; Barreto 2010; Zahra et al. 2006), researchers have also recognised the usefulness of the DCA (e.g., Borch and Madsen 2007; Weerawardena et al. 2007), including to study FBs (Chirico and Nordqvist 2010). The existing academic literature has highlighted various research gaps associated with DCs. One such limitation was that the DCA has been applied to examine FBs to a very limited extent (Jones et al. 2013). Another fundamental knowledge gap has arisen due to lack of qualitative research approaches being utilised to fully understand FBs (Fletcher et al. 2016).

In addressing these research gaps, the study proposed a framework grounded on the DCA (Fig. 1) to examine FBs’ adaptation to changes in the business environment from the perspective of 10 FBs operating in Western Australia. Despite the socioeconomic importance of FBs, for instance, in terms of domestic or international trade and gross domestic product, there has been a lack of academic studies focusing on this state’s FBs.

The findings clearly demonstrated that, to respond to the rapidly changing environment which they operated in, the participating FBs were fundamentally embracing innovation, to add value and to gain in efficiencies. Some have also utilised innovation to establish and strengthen networks with their supply chain or industry relationships. The DCs and DCA were manifested in various forms (Table 2). For instance, all three clusters of activities (Teece 2007, 2012), coupled with organisational heritage, “home-grown” capabilities, VRIN attributes, open culture, signature processes and idiosyncratic/tacit knowledge were observed in most cases. The results also supported findings made by Duran et al. (2016), who compared FBs with first-generation versus those with more than one generation, and found that, instead of being acquired within a short-term, DCs are built-up and developed over an extensive period. Furthermore, the progressive accumulation of knowledge, including tacit knowledge, expertise, skills, or as in the cases of Ely and Sam, strategically important assets (cold chain, cleaner sources of energy) were strongly associated with the VRIN attributes (Barney 1991).

While this research only focused on FBs, arguably, the findings and their associations with the DCA could be transferrable to other, non-family business environments. Indeed, some of the instruments the participating FBs were employing, including executing innovative/problem-solving strategies and initiatives that were knowledge-based, have been emphasised in other contexts (e.g., Piening and Salge 2015; Zheng et al. 2011).

Diehr and Wilhelm’s (2017) investigation of small and medium enterprises noticed that identifying various knowledge sources to adapt or generate services and products among firms are viewed as the pinnacle of a firm’s processes and represent more than mere routines. Moreover, three necessary processes were revealed in Diehr and Wilhelm’s (2017) research: the development of knowledge networks, solving customer issues, and acquiring absorptive capacity to integrate and understand customer knowledge. Importantly, these processes are linked to acquiring explicit and building tacit knowledge, and in turn are associated with other adaptive characteristics, including adding value, or diversifying, aligning with Duran et al.’s (2016) work. Thus, this study’s findings could provide valuable insights to other business contexts, including those considering the DCs and the DCA to understand firm adaptation and resilience.

### Implications

Given the critical role FBs play in many economies, finding practical ways to understand how FBs are identifying (sensing), accumulating, operationalising and maximising (seizing) valuable resources, alongside reinventing or extending their repertoire of resources (transforming) is crucial. From a practical perspective, one key implication is that, no matter how successful the business is, FB entrepreneurs must be prepared for continuous transformation and change. While there is evidence that some FBs may be prone to being static entities and therefore unwilling to change (Chirico and Nordqvist 2010), all the participating firms, regardless of their generational life cycle, were clearly involved in dynamic and constant change. Some of them had accumulated tacit/idiosyncratic knowledge throughout generations, while others possessed valuable and rare resources. Similarly, others were involved in signature processes, particularly through their strong interest and passion. However, all of them exhibited a drive for continuous learning, and perceived change as an inevitable, natural occurrence. In most cases, FBs were sensing and seizing opportunities by leveraging their own skills, engaging in innovation and continuous improvement.

The practical implications of this study are also intrinsically related to those of a theoretical perspective, and illustrated by the proposed framework. This ideology holds potential to help develop understanding and rigour based on the DCA, whose adoption in empirical FB research has been considered to a very limited extent (Jones et al. 2013). One fundamental theoretical implication is that, by illustrating the critical associations between sources of competitive advantage and the “backbone” represented by the clusters of activities (Teece 2007, 2012, 2014a), the framework can help elucidate the extent, and specific ways, in which FBs possess those sources and achieve competitive/sustained competitive advantage. By contributing to a deeper understanding of these associations, the framework can also help identify ways in which FBs may be engaged in continuous learning and renewal. Arguably, among other forms, such outcomes were demonstrated in practical ways, including through the establishment and strengthening of networks (e.g., Jennie, Marie, John), which is also related to FBs open culture, and to the accumulation of idiosyncratic and tacit knowledge.

Therefore, the framework could be considered by FBs as a tool to identify ways to achieve competitive advantage. Again, the recognition of key characteristics in the family firm, as identified in some of the cases (e.g., Jennie, Tim, or Ely) and suggested in Fig. 1, could be used as a road map for firm owners/managers to reflect upon. Moreover, firm owners/managers could follow the different sections of the framework to pinpoint what characteristics or traits their firms possess versus areas that need to be improved or developed in order to achieve optimal entrepreneurial performance, and gain or consolidate competitiveness. The ability to develop critical mass or structures that allow flexibility and adaptability may be a reasonable outcome through utilising the framework. Importantly, at the end of this reflection, owners/managers could also confirm whether their firm possesses characteristics or resources to continue learning or to renew, which again could be conducive to further firm competitiveness.

### Limitations and Future Research

While the study provides useful insights, it also has various limitations. First, although the majority of the sampled FBs had existed for two or more generations, amassed a wealth of knowledge and expertise, and were leaders in their industries and region, that only ten participated limits the generalisability of the study. Second, the study also lacks a component of diversity across different industries, with a predominance of FBs operating in the food growing, supplier, and manufacturing sectors. Third, despite operating in other Australian states as well as internationally, all FBs are currently based in Western Australia. Future research could address these limitations by gathering the perspectives of FBs in other Australian states, increasing the number of participants, and widen the research scope to include firms operating in other sectors/industries, thus, providing opportunities for comparative analyses.

Similarly, future research could consider a cross-country perspective, combining Australian FBs with those of other nations to produce comparisons. These propositions for future research could illuminate practitioners and academics to the nuanced and innovative ways in which FBs operating in different locations and industries are building their DCs and adapting. Finally, researchers could consider adopting, confirming/disconfirming, refining or further developing this study’s proposed framework. Given the limited adoption of the DCA to study FBs identified in the literature (Jones et al. 2013) and in this study, the future consideration and inclusion of this approach would add more rigour and depth to understand FBs’ adaptation.

## References

[CR1] ABS. (2001). *Small business in Australia, 2001*. Retrieved from http://www.abs.gov.au/ausstats/abs@.nsf/0/97452F3932F44031CA256C5B00027F19?OpenDocument.

[CR2] Allison TH, McKenny AF, Short JC (2014). Integrating time into family business research: Using random coefficient modeling to examine temporal influences on family firm ambidexterity. Family Business Review.

[CR3] Ambrosini V, Bowman C (2009). What are dynamic capabilities and are they a useful construct in strategic management?. International Journal of Management Reviews.

[CR4] Ambrosini V, Bowman C, Collier N (2009). Dynamic capabilities: An exploration of how firms renew their resource base. British Journal of Management.

[CR5] Arend RJ, Bromiley P (2009). Assessing the dynamic capabilities view: Spare change everyone?. Strategic Organization.

[CR6] Astrachan JH, Shanker MC (2003). Family businesses’ contribution to the US economy: A closer look. Family Business Review.

[CR7] Barney J (1991). Firm resources and sustained competitive advantage. Journal of Management.

[CR8] Barney JB (2001). Resource-based theories of competitive advantage: A ten-year retrospective on the resource-based view. Journal of Management.

[CR9] Barney JB (2001). Is the resource-based ‘view’ a useful perspective for strategic management research? Yes. Academy of Management Review.

[CR10] Barreto I (2010). Dynamic capabilities: A review of past research and an agenda for the future. Journal of Management.

[CR11] Benavides-Velasco CA, Quintana-García C, Guzmán-Parra VF (2013). Trends in family business research. Small Business Economics.

[CR12] Borch OJ, Madsen EL (2007). Dynamic capabilities facilitating innovative strategies in SMEs. International Journal of Technoentrepreneurship.

[CR13] Bowen GA (2008). Naturalistic inquiry and the saturation concept: A research note. Qualitative Research.

[CR14] Chirico F, Nordqvist M (2010). Dynamic capabilities and trans-generational value creation in family firms: The role of organizational culture. International Small Business Journal.

[CR15] Chirico F, Salvato C (2008). Knowledge integration and dynamic organizational adaptation in family firms. Family Business Review.

[CR16] Cope DG (2014). Computer-assisted qualitative data analysis software. Oncology Nursing Forum.

[CR17] Cucculelli M, Mannarino L, Pupo V, Ricotta F (2014). Owner-management, firm age, and productivity in Italian family firms. Journal of Small Business Management.

[CR18] Dalpiaz E, Tracey P, Phillips N (2014). Succession narratives in family business: The case of Alessi. Entrepreneurship Theory and Practice.

[CR19] Danes SM, Meraz AA, Landers AL (2016). Cultural meanings of resource management for Mexican–Americans. Journal of Family and Economic Issues.

[CR20] Diehr G, Wilhelm S (2017). Knowledge marketing: How can strategic customers be utilised for knowledge marketing in knowledge-intensive SMEs?. Knowledge Management Research and Practice.

[CR21] Distelberg B, Blow A (2010). The role of values and unity in family businesses. Journal of Family and Economic Issues.

[CR22] Dumas C (1992). Integrating the daughter into family business management. Entrepreneurship: Theory and Practice.

[CR23] Duran P, Kammerlander N, Van Essen M, Zellweger T (2016). Doing more with less: Innovation input and output in family firms. Academy of Management Journal.

[CR24] Eisenhardt KM (1989). Building theories from case study research. The Academy of Management Review.

[CR25] Eisenhardt Kathleen M., Martin Jeffrey A. (2000). Dynamic capabilities: what are they?. Strategic Management Journal.

[CR26] Engel RJ, Schutt RK (2005). The practice of research in social work.

[CR27] Fletcher D, De Massis A, Nordqvist M (2016). Qualitative research practices and family business scholarship: A review and future research agenda. Journal of Family Business Strategy.

[CR28] Gedajlovic E, Carney M, Chrisman JJ, Kellermanns FW (2012). The adolescence of family firm research: Taking stock and planning for the future. Journal of Management.

[CR29] Glover JL (2010). Capital usage in adverse situations: Applying Bourdieu’s theory of capital to family farm businesses. Journal of Family and Economic Issues.

[CR30] Graneheim UH, Lundman B (2004). Qualitative content analysis in nursing research: Concepts, procedures and measures to achieve trustworthiness. Nurse Education Today.

[CR31] Gratton, L., & Ghoshal, S. (2005). Beyond best practice. *MIT Sloan Management Review, 46*(3), 49–57. Retrieved from https://search.proquest.com/docview/224961808?accountid=12118.

[CR32] Gronn P (2002). Distributed leadership as a unit of analysis. The Leadership Quarterly.

[CR33] Handler WC (1989). Methodological issues and considerations in studying family businesses. Family Business Review.

[CR34] Hatum A, Pettigrew A (2004). Adaptation under environmental turmoil: Organizational flexibility in family-owned firms. Family Business Review.

[CR35] Helfat CE, Peteraf MA (2003). The dynamic resource-based view: Capability lifecycles. Strategic Management Journal.

[CR36] Helfat CE, Winter SG (2011). Untangling dynamic and operational capabilities: Strategy for the (N) ever-changing world. Strategic Management Journal.

[CR37] Howorth C, Rose M, Hamilton E, Westhead P (2010). Family firm diversity and development: An introduction. International Small Business Journal.

[CR38] Jones O, Ghobadian A, O’Regan N, Antcliff V (2013). Dynamic capabilities in a sixth-generation family firm: Entrepreneurship and the Bibby Line. Business History.

[CR39] Lee YG, Marshall MI (2013). Goal orientation and performance of family businesses. Journal of Family and Economic Issues.

[CR40] Marshall B, Cardon P, Poddar A, Fontenot R (2013). Does sample size matter in qualitative research?: A review of qualitative interviews in IS research. Journal of Computer Information Systems.

[CR41] Marshall MN (1996). Sampling for qualitative research. Family Practice.

[CR42] McDonald TM, Marshall MI (2018). Family business responses to household and business cash-flow problems. Journal of Family and Economic Issues.

[CR43] Memili E, Fang HC, Welsh DH (2015). Value creation and value appropriation in innovation process in publicly-traded family firms. Management Decision.

[CR44] Miller D, Breton-Miller L (2006). Family governance and firm performance: Agency, stewardship, and capabilities. Family Business Review.

[CR45] Morgan DL (1993). Qualitative content analysis: A guide to paths not taken. Qualitative Health Research.

[CR46] Muske G, Winter M (2001). An in-depth look at family cash-flow management practices. Journal of Family and Economic Issues.

[CR47] Nonaka I, Von Krogh G (2009). Perspective –Tacit knowledge and knowledge conversion: Controversy and advancement in organizational knowledge creation theory. Organization Science.

[CR48] O’Reilly M, Parker N (2013). ‘Unsatisfactory Saturation’: A critical exploration of the notion of saturated sample sizes in qualitative research. Qualitative Research.

[CR49] Palinkas LA, Horwitz SM, Green CA, Wisdom JP, Duan N, Hoagwood K (2015). Purposeful sampling for qualitative data collection and analysis in mixed method implementation research. Administration and Policy in Mental Health and Mental Health Services Research.

[CR50] Patton MQ (2015). Qualitative research and evaluation methods.

[CR51] Piening EP, Salge TO (2015). Understanding the antecedents, contingencies, and performance implications of process innovation: A dynamic capabilities perspective. Journal of Product Innovation Management.

[CR52] Poza EJ, Daugherty MS (2014). Family business.

[CR53] Prencipe A, Bar-Yosef S, Dekker HC (2014). Accounting research in family firms: Theoretical and empirical challenges. European Accounting Review.

[CR54] Priem RL, Alfano F (2016). Setting new directions for the management discipline through family business research. Journal of Family Business Strategy.

[CR55] Ramírez Solís ER, Baños Monroy VI, Rodríguez Aceves LA, Kellermanns FW, Hoy F (2017). Family business in Latin America. The Routledge companion to family business.

[CR56] Rosenblatt PC, de Mik L, Anderson RM, Johnson PA (1985). The family in business.

[CR57] Rothaermel FT, Hess AM (2007). Building dynamic capabilities: Innovation driven by individual-, firm-, and network-level effects. Organization Science.

[CR58] Schreier M (2012). Qualitative content analysis in practice.

[CR59] Sharma P, Chua JH (2013). Asian family enterprises and family business research. Asia Pacific Journal of Management.

[CR60] Sharma P, De Massis A, Gagne M (2014). Family business: A fertile ground for research on time, teams and positive organizational study. European Journal of Work and Organizational Psychology.

[CR61] Sirmon DG, Hitt MA (2003). Managing resources: Linking unique resources, management, and wealth creation in family firms. Entrepreneurship Theory and Practice.

[CR63] Teece D, Pisano G (1994). The dynamic capabilities of firms: An introduction. Industrial and Corporate Change.

[CR64] Teece DJ (2007). Explicating dynamic capabilities: The nature and microfoundations of (sustainable) enterprise performance. Strategic Management Journal.

[CR65] Teece DJ (2012). Dynamic capabilities: Routines versus entrepreneurial action. Journal of Management Studies.

[CR66] Teece DJ (2014). The foundations of enterprise performance: Dynamic and ordinary capabilities in an (economic) theory of firms. The Academy of Management Perspectives.

[CR67] Teece DJ (2014). A dynamic capabilities-based entrepreneurial theory of the multinational enterprise. Journal of International Business Studies.

[CR68] Teece DJ, Pisano G, Shuen A (1997). Dynamic capabilities and strategic management. Strategic Management Journal.

[CR69] Thomas DR (2006). A general inductive approach for analyzing qualitative evaluation data. American Journal of Evaluation.

[CR70] Tongco MDC (2007). Purposive sampling as a tool for informant selection. Ethnobotany Research and Applications.

[CR71] Vanpoucke E, Vereecke A, Wetzels M (2014). Developing supplier integration capabilities for sustainable competitive advantage: A dynamic capabilities approach. Journal of Operations Management.

[CR72] Wang Y (2016). Environmental dynamism, trust and dynamic capabilities of family businesses. International Journal of Entrepreneurial Behavior and Research.

[CR73] Weerawardena J, Mort GS, Liesch PW, Knight G (2007). Conceptualizing accelerated internationalization in the born global firm: A dynamic capabilities perspective. Journal of World Business.

[CR74] Xi JM, Kraus S, Filser M, Kellermanns FW (2015). Mapping the field of family business research: Past trends and future directions. International Entrepreneurship and Management Journal.

[CR75] Yin RK (2009). Case study research: Design and methods.

[CR76] Zahra SA (2016). Developing theory-grounded family business research: Some suggestions. Journal of Family Business Strategy.

[CR77] Zahra SA, Sapienza HJ, Davidsson P (2006). Entrepreneurship and dynamic capabilities: A review, model and research agenda. Journal of Management Studies.

[CR78] Zheng S, Zhang W, Wu X, Du J (2011). Knowledge-based dynamic capabilities and innovation in networked environments. Journal of Knowledge Management.

